# The mediating role of self-efficacy between activation and exercise compliance in patients undergoing arthroscopic rotator cuff repair

**DOI:** 10.1097/MD.0000000000044753

**Published:** 2025-10-03

**Authors:** Xi Ren, Litian Zhang

**Affiliations:** a School of Nursing, Inner Mongolia Medical University, Hohhot, Inner Mongolia, China; b Quality Management Department, The Second Affiliated Hospital of Inner Mongolia Medical University, Hohhot, Inner Mongolia, China.

**Keywords:** arthroscopic rotator cuff repair, exercise compliance, patient activation, self-efficacy mediating effect

## Abstract

This study examined the mediating role of self-efficacy between patient activation and exercise compliance in arthroscopic rotator cuff repair (ARCR) patients. Convenience sampling method was adopted to select 305 patient cases who underwent ARCR in the sports medicine department of a tertiary orthopedic specialty hospital in Inner Mongolia Autonomous Region from September 2024 to May 2025. A self-administered general information questionnaire, Patient Activation Measure-13 (PAM-13) scale, Rehabilitation Exercise Compliance Scale, and General Self-Efficacy Scale were used to investigate the patients. Pearson correlations were performed using SPSS 26.0 (Chicago) to analyze the relationship between the Patient Activation Measure-13, Rehabilitation Exercise Compliance Scale, and General Self-Efficacy Scale. Mediating effects were tested by Bootstrap. ARCR patient activation was positively correlated with exercise compliance; patient activation and self-efficacy were positively correlated; and self-efficacy and exercise compliance were positively correlated. The results of the mediation effect analysis showed that the mediation effect of self-efficacy between activation and exercise compliance in ARCR patients was 0.066, and the mediation effect accounted for 50% of the total effect. Self-efficacy partially mediates the effect between patient activation and exercise compliance. Healthcare professionals and family members should enhance encouragement and supervision of patients to strengthen their belief in recovery and self-confidence, thus promoting individuals to adopt positive health exercise behaviors.

## 1. Introduction

Rotator cuff injury (RCI) is the predominant cause of shoulder joint pain and dysfunction, comprising approximately 50% of all shoulder-related conditions.^[1]^ According to research statistics, the annual global incidence of RCI ranges between 200,000 and 300,000 cases.^[2]^ The prevalence of RCI increases with age. The proportion of people over 60 years old is 25%, and it accounts for as high as 50% among people over 80 years old.^[3,4]^ Conservative treatment is widely utilized for tendinopathic conditions or tendon tears where the tear size is <50% of the tendon thickness. The treatment modalities include physical therapy (e.g., ice application or electrical nerve stimulation), pharmacotherapy (e.g., nonsteroidal anti-inflammatory drugs or corticosteroid injections), and exercise therapy, among others.^[5–7]^ Studies have demonstrated that 85% of patients with small to medium-sized rotator cuff tears experience improvements in pain and joint function following 6 to 12 weeks of standardized treatment. However, conservative treatment yields limited efficacy in cases of massive rotator cuff tears or tears accompanied by significant tendon retraction.^[8,9]^ Currently, arthroscopic rotator cuff repair (ARCR) is recognized as the gold standard for addressing rotator cuff injuries, particularly when conservative treatments prove ineffective.^[10]^ Research indicates that 1.5% to 32.7% of patients develop postoperative shoulder joint stiffness, and over 35% of patients are unable to fully restore their shoulder joint function to preoperative levels.^[11–13]^

Exercise compliance refers to the consistent adherence by patients to the health recommendations provided by healthcare professionals, enabling them to engage in self-care behaviors that enhance clinical outcomes and quality of life.^[14]^ Poor exercise compliance may result in a range of complications, including tissue adhesion, joint stiffness, and muscle atrophy. Effective adherence to rehabilitation exercises is a critical factor in the treatment of RCI. However, the noncompliance rate for postoperative exercises among patients can be as high as 70%.^[15]^ Factors influencing patient exercise compliance are multifaceted, with self-efficacy and patient activation demonstrating significant positive effects on compliance.^[16,17]^

Self-efficacy pertains to an individual’s belief in their ability to competently and effectively perform a specific task within a given context.^[18]^ Higher levels of self-efficacy have been shown to positively influence exercise compliance. By enhancing self-efficacy, individuals are more likely to develop both intrinsic and extrinsic motivation, cultivate positive beliefs, and thereby improve their commitment to regular exercise.^[19]^ Patient activation refers to an individual’s possession of the knowledge, skills, and confidence necessary for managing their health condition and engaging in self-care practices.^[20,21]^ Patient activation has a positive effect on self-efficacy and improves individual self-efficacy by instructing participants to build self-confidence, set goals, and self-health management and maintenance programs.^[22,23]^ Research indicates that patient activation and exercise compliance are interrelated rather than independent constructs. Furthermore, patient activation plays a significant role in promoting exercise adherence.^[17]^

While patient activation, self-efficacy, and exercise compliance are known to be interrelated, the underlying mechanisms among these factors remain understudied – particularly in the context of RCI. This study aims to explore the mediating role of self-efficacy in activation and exercise compliance of patients after ARCR.

## 2. Methods

### 2.1. Study design

This cross-sectional study was conducted from September 2024 to May 2025.

### 2.2. Participants

We used convenience sampling to enroll 305 patients who underwent ARCR at a tertiary orthopedic hospital in Inner Mongolia from September 2024 to May 2025. The study protocol was approved by the Institutional Review Board of the Second Affiliated Hospital of Inner Mongolia Medical University (No:EFY20250103(06)). All participants provided informed consent.

### 2.3. Inclusion and exclusion criteria

Inclusion Criteria: Inclusion criteria: meet the diagnostic criteria in the 2019 RCI Guidelines^[24]^ and confirmed by MRI of the shoulder joint; conservative treatment(physical therapy combined with pharmaceutical intervention) for >3 months with poor or ineffective efficacy; unilateral RCI; patient’s age was 18 to 75 years old; Consciousness was clear, with normal communication and understanding ability. Exclusion criteria: patients with definite psychiatric disorders (e.g., schizophrenia, depression, etc) or severe communication impairments (e.g., aphasia, hearing impairment, or other conditions that prevent normal communication). have a history of shoulder joint surgery in the past; combined with other shoulder diseases, such as a history of shoulder fractures, shoulder dislocations, shoulder joint infections, rheumatoid arthritis, etc. Combined with serious diseases of the heart, liver, kidneys and nervous system, etc.

### 2.4. Sampling method

Based on Kendall’s principle,^[25]^ the required sample size should be 5 to 10 times the number of study variables. With 23 investigated variables and accounting for 20% potential invalid responses, the maximum required sample size was 276 participants. During the study, we distributed 320 questionnaires and obtained 305 valid responses, yielding a 95.3% response rate.

### 2.5. Investigation tools

#### 2.5.1. General information questionnaire

During the study design phase, a general information questionnaire was developed based on a literature review, clinical practice, and expert opinion. It included patient demographics and disease-related information.

#### 2.5.2. Patient Activation Measure-13

The Patient Activation Measure-13 was developed by Hibbard^[26]^ in 2004 to assess individuals’ willingness and ability to manage their health. In this study, we used the patient activation level scale, which was revised and Chineseized by Hong.^[27]^ The scale consists of 4 dimensions: cognition (2 entries), belief (2 entries), skill (6 entries), and action (3 entries), with a total of 13 entries. Each item was scored on a Likert 5-point scale from “not applicable to one’s own situation” to “strongly agree,” with a score of 0 to 4. The original total score of the scale ranged from 0 to 52, which was standardized to 0 to 100 after logarithmic transformation, and the higher the score, the higher the patient’s enthusiasm for self-health management. Patient activation was categorized into 4 activation stages: stage 1 was ≤ 47.0 points, stage 2 was 47.1 to 55.1 points, stage 3 was 55.2 to 67.0 points, and stage 4 was ≥ 67.1 points. The scale Cronbach alpha coefficient was 0.835.The scale Cronbach alpha coefficient in this study was 0.947.

#### 2.5.3. Rehabilitation Exercise Compliance Scale

The Rehabilitation Exercise Compliance Scale was developed by Xiao^[28]^ in 2020 and was used to analyze the compliance of rehabilitation exercise after RCI surgery. The scale is divided into 3 dimensions: physical exercise (6 items), postoperative precautions (4 items), and actively seeking advice (3 items), totaling 13 items. For each item, the answers of “can’t do it at all,” “can do it occasionally,” “can do it basically,” and “can do it completely” are scored 0, 1, 2, and 3 respectively. The higher the score, the better the compliance. The Cronbach’sα coefficient of the scale was 0.781. In this study, the Cronbach α coefficient of the scale was 0.912.

#### 2.5.4. General Self-Efficacy Scale

This study employed the General Self-Efficacy Scale revised and translated into Chinese by Wang^[29]^ to assess the enhancement of patients’ self-efficacy and its promotion of positive behaviors. The scale is a unidimensional one with 10 items, using a 4-point Likert scale ranging from “completely incorrect” to “completely correct,” corresponding to scores of 1 to 4, with a total score of 40. The higher the score, the stronger the individual’s self-efficacy level. The Cronbach α coefficient of the scale is 0.87. In this study, the Cronbach α coefficient of the scale was 0.876.

### 2.6. Exercise training after ARCR

All ARCR patients included in this study followed a standardized rehabilitation protocol jointly developed by the Sports Medicine Center and the Rehabilitation Department of our hospital. The entire rehabilitation training process was guided by professional nurses and rehabilitation therapists, and was generally divided into 4 phases to guide patients through rehabilitation training.^[30]^ Phase 1 (1 Day to 6 Weeks Postoperatively) focuses on passive range of motion exercises: hand grasping exercises and wrist and elbow joint exercises are initiated immediately on the first postoperative day (10–20 repetitions per set, 3–5 sets per day); pendulum exercises are started at 1 week postoperatively (10 repetitions per set, 3 sets per day); table slide forward flexion, table slide abduction, and passive internal/external rotation exercises are introduced from 2 to 3 weeks postoperatively (15–30 repetitions per set, 2–3 sessions per day); and scapular stabilization exercises are conducted from 3 to 6 weeks postoperatively (10–15 repetitions per set, 2–3 sessions per day). For this phase, it is important to note that the shoulder joint range of motion should not exceed 120° of forward flexion and 30° of passive internal/external rotation, with all movements beyond this range and active shoulder joint movements prohibited. Phase 2 (6 to 12 Weeks Postoperatively) involves a gradual transition from active-assisted training to active training: active-assisted training was initiated from 6 to 9 weeks postoperatively, focusing primarily on active-assisted forward flexion, abduction, internal rotation, and external rotation exercises (15–20 repetitions per set, 2–3 sessions per day), with tools such as sticks and pulleys allowed for assistance during movements and the range of motion maintained at a maximum of 160° of forward flexion and 45° of external rotation; from 9 to 12 weeks postoperatively, active training was started, emphasizing active external rotation, abduction, and external rotation exercises (15–20 repetitions per set, 2–3 sessions per day), where patients were required to perform shoulder movements at all angles within the horizontal plane to the best of their ability, provided that no pain was experienced. Phase 3 (12 to 16 Weeks Postoperatively) consists of resistance training, which focuses primarily on isometric push-pull training and isometric internal/external rotation training (10–15 repetitions per set, 2–3 sessions per day), and for muscle strength training within this phase, the principle of low load and high repetition was strictly followed. Phase 4 (16 to 24 Weeks Postoperatively) focuses on functional recovery training, while continuing the resistance training from the previous phase; 2 types of stability training – stable ball-holding training and closed kinetic chain training – are implemented to enhance rotator cuff strength, and concurrently, exercises targeting shoulder joint coordination and flexibility are conducted.

### 2.7. Data collection method

A survey team was set up, which consisted of 1 head nurse of a sports medicine center, 2 charge nurses, and 2 rehabilitation therapists. All the aforementioned members were part of the clinical team from the service departments where the patients involved in the study received their treatment, and they were responsible for the distribution and collection of questionnaires. Before distributing the questionnaires, the team members were trained, and all of them participated in this study after passing the examination to ensure the accuracy and consistency of the collection process. Before the study subjects filled in the questionnaire, the researcher used a unified guide to explain the requirements for filling in the questionnaire, and if the patients had any questions during the process of filling in the questionnaire, the researcher used a consistent language to explain the questionnaire, and the questionnaire was retrieved for on-the-spot checking, and if there were any missing items, the patients were asked to fill in the questionnaires again with additional information and retrieve them after explanation. Finally, the questionnaire collation and data analysis were conducted by researchers who were not involved in the distribution or collection of the questionnaires.

### 2.8. Statistical method

All the collected data were uniformly numbered, and the data were entered using Excel software using the 2-person entry method, and some of the data were extracted for review after completion to ensure the accuracy of the entered data. SPSS26.0 was used for statistical analysis, and the count data were expressed as frequency counts and constitutive ratios, and the measurement data were expressed as mean ± standard deviation. Correlations of patient activation, exercise adherence and self-efficacy were analyzed by Pearson correlation analysis; mediated effects were tested using the Bootstrap program, and significance was tested by applying confidence interval estimation, with 95% confidence intervals that did not contain 0 as the presence of a mediating effect. A difference of *P *< .05 was considered statistically significant.

## 3. Result

### 3.1. Common method deviation test

We assessed potential common method bias using Harman single-factor test.^[31]^ Results showed 5 factors with eigenvalues >1. The first factor accounted for 33.725% of the total variance, below the 40% threshold, indicating no significant common method bias in our data.

### 3.2. General information

A total of 305 patients with ARCR were enrolled in this study. Regarding age distribution, participants aged 45 to 59 accounted for the highest proportion (48.2%), followed by those aged 60 and above (46.6%). In terms of gender composition, males accounted for a higher proportion (53.4%). For residential location, the largest number of participants resided in provincial capital cities (43%). Regarding marital status, the majority were married (79.3%). In terms of educational attainment, participants with junior high school education accounted for the highest proportion (34.4%), and the largest group of participants had a monthly household income of less than 3000 Renminbi (RMB). In terms of clinical characteristics, most patients had acute rotator cuff injuries, with the right shoulder being the predominant site of injury, and the injury duration was within 3 months. Regarding postoperative rehabilitation characteristics, spouses served as the main caregivers (45.9%); 41.0% of patients perceived a moderate level of difficulty in rehabilitation exercises; and family members were the primary source of rehabilitation support (43.0%)(Table 1).

**Table 1:** General information of patients (n = 305).

Project	Numbers	Ratio (%)	Compliance score (score)	*t/F*	*P*
Age (year)					
18–44	16	5.2	20.75 ± 6.74	26.197	<**.001**
45–59	147	48.2	24.78 ± 5.36		
60–75	142	46.6	20.21 ± 5.4		
Gender					
Male	163	53.4	22.8 ± 6.23	1.148	.252
Female	142	46.6	22.03 ± 5.47		
Residence					
Provincial capital	131	43	22.79 ± 6.13	0.637	.592
County/District	86	28.2	21.91 ± 5.76		
Township/Town	36	11.8	23.11 ± 5.47		
Rural area	52	17	22 ± 5.82		
Occupation					
Farmer	76	24.9	21.09 ± 5.17	1.963	.12
Retirement	94	30.8	22.56 ± 6		
Employed	83	27.2	23.07 ± 6.36		
Other	52	17	23.19 ± 5.72		
Marital status					
Unmarried	4	1.3	24 ± 10.42	5.969	<**.001**
Married	242	79.3	23.1 ± 5.88		
Divorce	30	9.8	20.27 ± 4.39		
Widowed	29	9.5	19.03 ± 5.05		
Level of education					
Primary school and below	75	24.6	19.45 ± 4.39	12.67	<**.001**
Junior high school	105	34.4	22.95 ± 6.49		
High school	81	26.6	24.85 ± 3.1		
College degree or above	44	14.4	21.89 ± 8.05		
Monthly household income					
<3000	125	41	20.44 ± 5.27	16.086	<**.001**
3000–5000	114	37.4	23.11 ± 5.59		
>5000	66	21.6	25.09 ± 6.26		
Health care type					
Medical insurance for urban residents	87	28.5	23.83 ± 6.45	4.196	**.006**
New rural cooperative medical system	95	31.1	22.95 ± 5.73		
At one’s own expense	79	25.9	21 ± 4.73		
Others (Employee insurance, commercial insurance)	44	14.4	21.2 ± 6.29		
Disease course					
<3 mo	154	50.5	21.64 ± 5.43	3.026	**.03**
3–6 mo	91	29.8	22.59 ± 5.54		
7 mo-1 y	40	13.1	24.13 ± 6.71		
>1 y	20	6.6	24.6 ± 7.92		
Damaged part					
Left side	119	39	22.86 ± 6.02	0.984	.326
Right side	186	61	22.18 ± 5.8		
Caregiver					
Oneself	94	30.8	21.05 ± 5.41	6.635	**.002**
Spouse	140	45.9	23.72 ± 5.9		
Children	71	23.3	21.76 ± 6.01		
Etiology					
Acute injury	193	63.3	22.4 ± 5.85	−0.15	.881
Degenerative lesions	112	36.7	22.51 ± 5.97		
Type of brace to wear					
Neck and wrist sling	82	26.9	22.13 ± 5.19	-0.554	.58
Outreach package	223	73.1	22.56 ± 6.13		
Self-perceived exercise intensity					
Greater difficulty	85	27.9	20.79 ± 5.47	11.235	<**.001**
Average difficulty	125	41	21.9 ± 5.33		
No difficulty	95	31.1	24.64 ± 6.33		
Rehabilitation encouragement					
Rehabilitation therapist	84	27.5	23.49 ± 6.52	6.269	**.002**
Family members	131	43	23 ± 5.89		
None	90	29.5	20.66 ± 4.83		

GSES = General Self-Efficacy Scale, PAM-13 = Patient Activation Measure-13.

### 3.3. Exercise compliance, patient activation, and self-efficacy scores in ARCR patients

The total exercise compliance score was (22.44 ± 5.89), physical exercise dimension score (9.89 ± 3.02), postoperative precautions dimension score (7.3 ± 1.88), and active advice seeking dimension score (5.25 ± 1.7). The trans-standardized total score of patient activation was (51.79 ± 14.92), which was in the second stage overall, with 130 cases (42.6%) in the first level, 82 cases (26.9%) in the second level, 58 cases (19%) in the third level, and 35 cases (11.5%) in the fourth level. The self-efficacy dimension score (24.5 ± 5.3) (Table 2).

**Table 2:** Scores of patient activation, exercise compliance, and self-efficacy in arthroscopic rotator cuff repair patients (n = 305).

Scale	Number of items	Total score ($\bar{x}\pm s$)	Average score ($\bar{x}\pm s$)
PAM-13 scores			
Total	13	51.79 ± 14.92	3.98 ± 1.15
Cognition	2		
Skill	6		
Action	3		
Belief	2		
Exercise Compliance scores			
Total	13	22.44 ± 5.89	1.73 ± 0.45
Physical exercise	6	9.89 ± 3.02	1.65 ± 0.5
Postoperative precautions	4	7.3 ± 1.88	1.83 ± 0.47
Actively seeking advice	3	5.25 ± 1.7	1.75 ± 0.57
GSES scores			
Total	10	24.5 ± 5.3	2.45 ± 0.53

GSES = General Self-Efficacy Scale, PAM-13 = Patient Activation Measure-13.

### 3.4. Factors influencing exercise compliance after ARCR

The results of the univariate analysis showed that the scores of patients’ exercise compliance were significantly different in terms of age, educational level, marital status, monthly income, payment method, disease duration, caregiver, perceived difficulty of exercise, and rehabilitation encouragement and assistance (*P *< .05) (Table 1).

### 3.5. Correlation analysis of exercise compliance patient activation, and self-efficacy in ARCR patients

**Figure 1.**
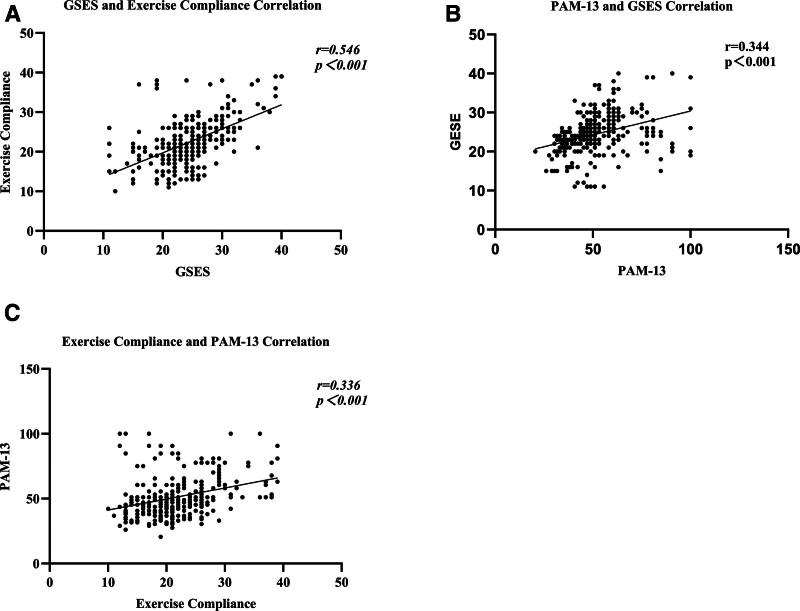
(A) GSES and exercise compliance correlation; (B) PAM-13 and GSES correlation; (C) exercise compliance and PAM-13 correlation. GSES = General Self-Efficacy Scale, PAM-13 = Patient Activation Measure-13.

The results of Pearson correlation analysis showed that patients’ exercise compliance was significantly positively correlated with patient activation and self-efficacy. Patient activation was positively correlated with exercise compliance (*P *< .05); patient activation was positively correlated with self-efficacy (*P *< .05); self-efficacy was positively correlated with exercise compliance (*P* < .05) (Table 3 and Fig. 1).

**Table 3:** Correlation analysis of exercise compliance, patient activation, and self-efficacy in arthroscopic rotator cuff repair patients (n = 305).

Items	Exercise compliance	GSES	PAM-13
Exercise compliance	1	–	–
GSES	0.546*	1	–
PAM-13	0.336*	0.344*	1

GSES = General Self-Efficacy Scale, PAM-13 = Patient Activation Measure-13.

* *P *< .001.

### 3.6. Mediating effect of self-efficacy between exercise compliance and patient activation in ARCR patients

**Figure 2.**
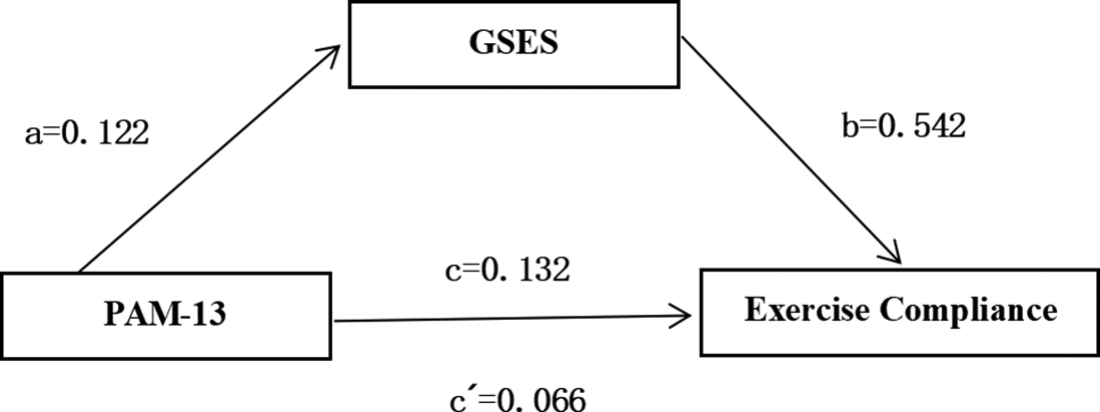
The mediating role of self-efficacy in patient activation and exercise compliance.

In this study, the mediating role of self-efficacy between patient activation and exercise compliance was examined using Model 4 of the Process Bootstrap program, with standardized PAM score as the independent variable, exercise compliance score as the dependent variable, and self-efficacy score as the mediating variable. The results showed that the total effect of standardized PAM score on exercise compliance score was 0.132; the effect value of standardized PAM score on exercise compliance score was 0.066 (*t* = 3.329, *P *< .001); the effect value of standardized PAM score on self-efficacy score was 0.122 (t = 6.373, *P *< .001); and the effect value of self-efficacy score on exercise compliance score was 0.542. score effect value was 0.542 (*t* = 9.68, *P *< .001); self-efficacy partially mediated the effect between standardized PAM scores and exercise compliance scores, with an effect value of 0.066, and the mediating effect percentage was 50% (Table 4 and Fig. 2).

**Table 4:** Mediating effect of self-efficacy between exercise compliance and patient activation in arthroscopic rotator cuff repair patients.

Effect	Path	B	SE	*P*	95% CI	Percent (%)
Direct effect	PAM→exercise compliance	0.066	0.019	<.001	0.090–0.174	50
Indirect effect	PAM→GSES→ exercise compliance	0.066	0.015	<.001	0.038–0.098	50
Total effect	PAM→exercise compliance	0.132	0.021	<.001	0.090–0.335	100

GSES = General Self-Efficacy Scale, PAM-13 = Patient Activation Measure-13.

## 4. Discussion

### 4.1. Exercise compliance levels were moderate in ARCR patients

The results of this study showed that the exercise compliance score of rotator cuff repair patients was (22.44 ± 5.89), which was at a moderate level, reflecting that the patients’ motivation to participate in functional joint exercises needs to be improved, and this study was slightly higher than the results of She et al study.^[32]^ The key reason for the score similarity lies in both studies’ adoption of phased standardized protocols, which established clear exercise types and intensity standards for different post-ARCR periods based on the physiological characteristics of rotator cuff tissue healing. Decomposing rehabilitation training into actionable phased tasks reduced patients’ uncertainty about exercise training, laying the foundation for improved exercise adherence. Previous studies^[33,34]^ have demonstrated that compared with conventional rehabilitation training, phased standardized training develops rehabilitation protocols based on patients’ specific conditions, providing a clear implementation framework to reduce resistance caused by ambiguous training procedures. Additionally, with the assistance of rehabilitation therapists, it improves the completion rate and quality of patients’ training movements. Compared with other studies,^[28]^ the score of the physical exercise dimension in exercise adherence was lower in this study. The reasons for this phenomenon can be explored from multiple aspects: on the 1 hand, most patients participating in this study were middle-aged and elderly individuals with relatively low educational levels. Due to the gradual increase in age, this group may experience age-related physiological cognitive decline (such as memory loss and decreased comprehension ability) and lack of rehabilitation knowledge, which leads to reduced acceptance and implementation of exercise plans and difficulty in mastering standardized rehabilitation exercise methods.^[35,36]^ On the other hand, different age groups showed significant differences in physical tolerance and movement execution accuracy for rehabilitation training at various post-ARCR stages. In this study, patients aged 18–44 had moderate exercise adherence. Despite their notable advantages in learning efficiency, muscle strength recovery, and rehabilitation tolerance – quickly mastering tool-assisted joint training (e.g., with sticks, pulleys) and easily meeting the low-load, high-repetition demands of resistance training and complex functional recovery movements^[3,37]^ – this group is mostly in critical phases of family care and career development. This limits their time and energy for rehabilitation, leading to frequent training interruptions or simplified implementation. Compared with patients aged 18–44 years, those aged 45–60 years demonstrated better adherence. The reasons for this are as follows: first, this age group has stronger adaptability to the training protocol, and the types of training movements are directly related to patients’ daily life or work needs, making it easier for patients to persist in training and ensure training continuity; second, the training intensity and frequency are well-aligned with the time availability of patients in this age group, leading to fewer training interruptions due to work conflicts and making it easier to develop consistent training habits. However, patients aged 60–75 years had the lowest exercise adherence. During the early passive training phase, they could basically cooperate with the implementation, as the movements were relatively basic and simple, with low physical and cognitive requirements. However, in the resistance training and functional recovery phases, the training movements were complex – involving multi-joint coordinated movements and requiring relatively high sustained physical strength – making it difficult for patients to master the coherent process and thus reducing their training willingness.^[38,39]^

Economic income and medical insurance significantly impact patients’ participation in rehabilitation training.^[16,40]^ In the study on socioeconomic status and ARCR access, Gatto et al.^[41]^ found that patients with low socioeconomic status had fewer continuous care opportunities – resulting in poor mastery of rehabilitation protocols and significantly higher training interruption rates. Previous studies^[42]^ noted only 27% of Medicaid patients could access orthopedic appointments and consultations. This study showed that individuals with better socioeconomic status and medical insurance had relatively stronger willingness and motivation to participate in training. This can be explained by the inherent nature of rotator cuff rehabilitation: it is long-term and complex, requiring substantial time and effort. In contrast, patients with higher income and medical insurance do not need to worry about rehabilitation costs, better ensuring training continuity. Meanwhile, family support is especially important for patients’ rehabilitation and exercise.^[43]^ Nayak et al.^[16]^ conducted qualitative interviews with 13 patients who received outpatient physical therapy after ARCR. They found that physical therapists, as the core guiding entities of training, motivated patients to engage in training by developing personalized training protocols, correcting movement deviations, and addressing questions or difficulties. Meanwhile, family members ensured patients’ training enthusiasm through emotional encouragement and daily supervision.

### 4.2. Correlation analysis of exercise compliance, patient activation, and self-efficacy in ARCR patients

The results of this study showed a positive correlation between patient activation and self-efficacy, similar to that reported by Tang et al^[44]^ and Su et al^[45]^ studies. When an individual’s activation is high, it indicates that he or she is willing and able to participate in rehabilitation training. Under the guidance of physical therapists, the development of standardized exercise training checklists and training goals translates this active participation into a clear rehabilitation pathway. The positive feedback from such goal-oriented joint function recovery can inversely strengthen patients’ rehabilitation beliefs, thereby improving their self-efficacy.

Self-efficacy and exercise compliance were positively correlated, which is consistent with the findings of Burns et al.^[46]^ The negative emotions and low self-efficacy of patients with RCI during rehabilitation exercises due to joint pain, fear of exercise and lack of social support significantly affected patients’ adherence to exercise, which led to unsatisfactory recovery of joint function. It can thus be seen that self-efficacy serves as a key bridge connecting individuals’ psychological states and health behaviors. Individuals with low self-efficacy are more likely to perceive pain after ARCR as a sign of tissue damage and view complex training movements as insurmountable challenges. Such misconceptions lead patients to avoid rehabilitation training, thereby affecting their exercise participation. In contrast, patients with high self-efficacy perceive pain as motivation for training and establish phased rehabilitation goals under the guidance of physical therapists.

There was a positive correlation between patient activation and exercise adherence, consistent with the study by Qu et al^[17]^ It takes 3 to 6 months of standardized exercise training to resume daily activities after RCI, during which patients need to have the knowledge, skills and confidence in self-management of their health in order to activate patients’ beliefs about exercise, which will lead to the generation and maintenance of exercise behaviors, and thus improve exercise compliance. Patients with high activation not only have the willingness to actively engage in rehabilitation training but also possess problem-solving abilities, enabling them to distinguish between normal responses and abnormal signals during training. When experiencing increased pain after training, they proactively consult rehabilitation therapists to adjust the training protocol instead of giving up directly. It is precisely this ability that helps them overcome the frustration caused by slow joint recovery, transforming passive implementation into active training.

### 4.3. Self-efficacy partially mediates the relationship between exercise compliance and patient activation

The results of the mediated effects modeling in this study showed that self-efficacy partially mediated between patient activation and exercise compliance, with the mediating effect accounting for 50% of the total effect. The conclusions suggest that patient activation can directly influence exercise compliance and can indirectly influence adherence through self-efficacy. This aligns with the core role of self-efficacy in behavior change as outlined in Bandura Social Cognitive Theory. This finding further supports that self-efficacy is likely a key link in translating psychological motivation into behavioral decisions during postoperative rehabilitation training. Individuals with high self-efficacy were more positively engaged in rehabilitation exercise therapy, better able to avoid the occurrence of injurious health behaviors, and had higher expectations of joint function recovery following exercise therapy. It is this positive expectation that further strengthens rehabilitation beliefs and thus significantly enhances exercise compliance. Therefore, physical therapists and family members should strengthen the encouragement and supervision of patients to promote individuals to adopt positive health behaviors. Even family members and patients can personally participate in and formulate exercise and workout plans, so that the exercise program can be more in line with the actual condition of the patient’s disease, thus maximizing the patient’s subjective initiative. Therefore, physical therapists and family members should prioritize fostering patients’ self-efficacy. Guiding patients to set behavioral goals, develop plans, and build internal competencies better activates internal motivation than relying solely on external support to drive behavioral change. Meanwhile, strengthening patient encouragement and supervision helps consolidate positive behaviors. Additionally, involving both families and patients in creating personalized training protocols ensures alignment with patients’ actual disease conditions, maximizing their subjective initiative. To lay the foundation for improved exercise adherence, a systematic self-efficacy enhancement pathway should be established, integrating 3 key components: cultivating internal capabilities, providing external support, and activating patient engagement.

### 4.4. Limitations

This study also has the following limitations: the type of this study is a cross-sectional study, and it is not yet possible to determine the causal relationship between variables. The research subjects were selected only in a third-class orthopedic hospital in Inner Mongolia Autonomous Region, and the sample selection was not representative to a certain extent, and further multi-center, cross-regional, and large-sample studies can be conducted in the future. The survey tool is mainly evaluated by patients relying on their subjective will, and more objective indicators should be adopted for evaluation in the future.

## 5. Conclusion

This study verifies the mediating effect of self-efficacy in exercise compliance and patient activation in rotator cuff repair patients, which is of positive significance in enhancing patients’ exercise compliance. Healthcare professionals and family members should strengthen the care and encouragement for patients, improve self-confidence in exercise rehabilitation, stimulate self-efficacy, promote the maintenance of exercise compliance, and achieve the goal of joint function recovery.

## Author contributions

**Conceptualization:** Xi Ren.

**Data curation:** Litian Zhang.

**Formal analysis:** Xi Ren.

**Investigation:** Litian Zhang.

**Methodology:** Xi Ren.

**Project administration:** Litian Zhang.

**Resources:** Xi Ren.

**Software:** Xi Ren.

**Supervision:** Litian Zhang.

**Writing – original draft:** Xi Ren.

**Writing – review & editing:** Litian Zhang.
